# Remarkably lower consumption of antidepressants in Serbia in comparison with Finland

**DOI:** 10.1186/2050-6511-13-S1-A5

**Published:** 2012-09-17

**Authors:** Maja Stojančević, Milica Paut Kusturica, Bojan Stanimirov, Nebojša Pavlović, Zdenko Tomić, Ana Sabo, Momir Mikov

**Affiliations:** 1 Department of Pharmacology, Toxicology and Clinical Pharmacology, Faculty of Medicine, University of Novi Sad, 21000 Novi Sad, Serbia

## Background

Depression is an important health problem worldwide due to significant disability that it causes, reduction of quality of life, loss of work days, and even suicide. The aim of our survey was to evaluate the overall utilization and pattern of use of antidepressants in Serbia in a comparison with Finland in 2010 and to propose appropriate interventions in Serbia on the basis of the results obtained.

## Methods

The data on utilization of antidepressant drugs (ATC group N06) for 2010 were retrieved from the annual reports of relevent public institutions in Serbia and Finland. The ATC/DDD methodology was applied and the results were expressed in defined daily doses per 1000 inhabitants per day (DID). As an indicator of the quality of drug prescribing, the Drug Utilization 90% (DU90%) method was used.

## Results

An overall antidepressant consumption in 2010 appeared to be 6-fold lower in Serbia (11.7 DID) in comparison with Finland (68.8 DID). Selective serotonine reuptake inhibitors (SSRIs) accounted for the majority of antidepressant utilization in both countries (73.8% for Serbia and 63.9% for Finland). Apart from that, the pattern of utilization of the most frequently used SSRI drugs was different between these countries. Sertraline accounted for the highest share in Serbia (5.6 DID) while citalopram and escitalopram are generally the most widely used drugs for the treatment of depression in Finland (17.5 DID and 11.8 DID respectively). In our country, the group of nonselective monoamine reuptake inhibitors (13.1%) took the second place that makes a notable difference in comparison with Finland where the second-ranked group was other antidepressants (N06AX) (29.2%).

## Conclusions

The differences between selected countries in antidepressant utilization are partly consequential to different socioeconomic and health policy factors. The considerably lower utilization of antidepressants in Serbia implies possible underdiagnosing of affective disorders in general practice. To reduce the serious consequences that may be caused in that way, the early diagnosis and timely, adequate and effective management and treatment of depression is essential.

